# Evidence from a Longitudinal Photovoice and Interview Assessment with Congolese Refugee Women in the Midwestern United States

**DOI:** 10.1089/heq.2020.0121

**Published:** 2021-09-08

**Authors:** Shannon McMorrow, Jyotika Saksena

**Affiliations:** ^1^ School of Interdisciplinary Health Programs, Western Michigan University, Kalamazoo, Michigan, USA.; ^2^ Department of History and Political Science, University of Indianapolis, Indianapolis, Indiana, USA.

**Keywords:** photovoice, refugee health, immigrant health, women's health, Democratic Republic of Congo, qualitative research

## Abstract

**Purpose:** Refugees from the Democratic Republic of Congo have rapidly increased since 2016 and are growing to represent one of the top refugee groups in the United States. They are at high risk for health inequities, yet, there is limited qualitative research exploring the health needs, assets, and experiences of this group and even less longitudinal research. In addition, women refugees are understudied across all global contexts. Therefore, the purpose of this study was to conduct longitudinal qualitative research to provide rich contextual data on health and integration experiences of Congolese refugee women when they were newly resettled in 2016 and 3 years later in 2019.

**Methods:** We conducted photovoice and interviews with 16 women in March through May of 2016 and 10 of the same women in March and April of 2019.

**Results:** Women chose and discussed photos revealing a multitude of assets and needs spanning 2016 and 2019. Experiences with nutrition and food security were illuminated and are the focus of this article. Two major themes were access to food in contrast with availability of abundance of food in the United States and concern about what constitutes healthy food in the United States contrasted with accessing healthy and culturally appropriate food in the United States. Findings highlight strength bases of nutritional knowledge, attitudes, and skills as well as a strong social network aiding food security demonstrated by the Congolese refugee women in the study, offering an opportunity to shift to an assets and strength-based approach. Findings also note risk of food insecurity linked to barriers to employment and sociohistorical reflection on living with food shortages before migration to the United States that should be considered as providers strive to provide culturally relevant care.

**Conclusion:** Findings offer contextual data for health care providers and public health professionals to improve nutritional health promotion and food security support for this population. Overlooking nuanced structural barriers may lead to providers perpetuating health inequities for this population.

## Introduction

Humanitarian crises in the Democratic Republic of Congo (DRC) have continued for decades, causing mass displacement of people seeking refuge worldwide including the United States.^1^ Since 2016, Congolese refugees have represented the largest proportion of refugees resettled in the United States.^2,3^ A baseline of epidemiological health information is available about priority health needs of recent Congolese refugees such as malaria, mental health, parasitic infections, and sexual and gender-based violence.^4^ However, a more comprehensive evidence base that includes qualitative data to add a deeper picture of health needs is limited. There is an urgent window of opportunity for public health and health care systems to support this growing population who face health inequities such as higher risk for noncommunicable diseases, access to health insurance and care, language barriers, trauma history,^4^ and potential discrimination toward immigrants and racism toward Black people.^5^

The aforementioned health inequities for Congolese refugees are amplified for women due to pervasive gender inequities.^4,6^ Several researchers have called for more qualitative and participatory research to investigate experiences of refugee women with the ultimate aim of building health equity.^7–14^ However, the body of research focusing specifically on Congolese refugee women in settings in the United States is limited, with a notable gap in longitudinal evidence beyond the initial resettlement process.^6,11,15–20^

Our study started in 2016 as a community-based participatory research project (CBPR) in partnership with a refugee resettlement agency in a large midwestern city. In keeping with the principles of CBPR, the overarching aim was determined in consultation with the resettlement agency,^21^ which was to conduct an inductive qualitative examination to better understand health and integration experiences for Congolese refugee women. The study evolved to include longitudinal data collection at two points in 2016 and 2019. One major finding related to health that emerged over both points in time centered on nutrition and food security. The focus of this article is those results and related recommendations.

Parasitic infections and malaria are priority health conditions for Congolese refugees, both of which are impacted by nutrition.^4^ Risk is lower for wasting for this population than for other refugee groups. On the other end of the spectrum, evidence suggests that the longer Congolese refugees are resettled in the United States, the more the risk for conditions such as hypertension and diabetes.^4,22^ Data for type 2 diabetes and other nutrition-related chronic diseases for Congolese immigrants in the United States are limited. Prevalence of type 2 diabetes for the DRC was 6% in 2019, suggesting potential risk for Congolese refugees in the U.S context where they likely to face dietary acculturation, prior food scarcity and insecurity, and a health care system with multiple access barriers.^19,23,24^

Higher rates of household food insecurity have been documented for Sub-Saharan African refugees compared with other refugee groups and the general population,^19,25,26^ with estimates of 53–85% of food insecurity for this population.^19^ Yet, limited literature is available to public health or health care providers seeking evidence for better understanding of sociocultural contextual factors surrounding food security specifically for Congolese refugees in the U.S. context.^19,27–29^ A recent study examined challenges and assets to food security for Congolese and Burundian refugee women in the southeastern United States,^19^ and culminated in participatory adaptation of a culturally relevant cooking curriculum.^27,28^ Our study builds on the work of McElrone et al. by exploration in another region of the United States (midwest) and by adding longitudinal evidence to add additional historical, social, and cultural context to inform interventions designed to improve nutritional health promotion and food support interventions. Findings from this longitudinal study illustrate needs, assets, and potential points of intervention for nutritional health promotion and food security promotion to both build on existing assets and meet the unique needs of Congolese refugee women in the context of the midwestern United States.

## Methods

This qualitative study was initiated as a CBPR project with a refugee resettlement agency. The agency took the lead in collaboration for funding, choosing the study population, and providing staff support for the project. The sample was intentionally small aligning with norms for qualitative research to conduct in-depth examination,^30^ and with recommendations for sample sizes in photovoice research.^31,32^ Photovoice was chosen for its history as a participatory research method in women's global health, because it doubles as a health promotion intervention with potential for participant empowerment, and potential to promote health equity for vulnerable populations such as refugees.^33–36^ The institutional review boards of both authors approved all study procedures.

### Recruitment and participants

Recruitment entailed posting flyers at the refugee resettlement agency and purposive recruitment by a linguistically and culturally appropriate research team member. Twenty-one women who met inclusion criteria of female, 18 years or older, and self-identified as a Congolese refugee were recruited: 16 were retained in 2010 and 10 were retained in 2019. One participant had moved to another city and the other four declined due to work conflicts or lack of interest.

There was diversity in marital status, age, and education across the sample, including women who were single, married, and widowed, of ages 25 to 57 years, and women with no formal education and those who completed 13 years of schooling in Congo. In 2019, 90% of the women (*n*=9) had health insurance through the Healthy Indiana Plan 2.0, Indiana's version of Medicaid. When asked to provide self-ranking of their health, 30% noted “good,” 50% noted “fair,” and 10% noted “poor.” Of the 10 participants retained at both points of data collection, there was a negative change in employment. In 2016, 70% had part-time or full-time employment with 20% qualifying for disability and 10% not working. By 2019, the same two participants remained on disability, an additional participant was out of the workforce due to a severe workplace injury, one participant reported working, and the remaining six were not employed.

### Research procedures

Photovoice and semistructured interviews were conducted in both 2016 and 2019. Procedures in 2016 included interviews conducted at the participant's preferred location, mostly homes, before and after the photovoice sessions, and six 3-h long community-based photovoice sessions in April and May.^11,17,18^ Funding and time were reduced in 2019, so this resulted in abbreviated procedures for implementation. The interview component was shorter and occurred in a church community room before the first photovoice meeting. Participants then participated in two 3-h long photovoice sessions in March and April.

The majority of photovoice procedures remained consistent across 2016 and 2019. These included three of the four original women research team members; the photo assignment to take photos with study-provided digital cameras in their homes and communities of what made them feel happy, sad, or surprised about life in the United States and anything perceived to help or hinder their health and/or health care access; intentionally conducting photovoice meetings in community-based spaces; providing childcare; cultural tailoring through sharing east African food and music; interpretation processes with oral translation of questions from English to Kinyarwanda or Swahili, participants answer in their preferred language, and translation back to English; provision of a $20 gift card at the end of each session; and audiorecording of photovoice sessions with consent. All photovoice sessions were ∼3 h in length and were facilitated as modified focus groups following recommended photovoice procedures.^31,34^ Implementation differences between 2016 and 2019 were the number of photovoice sessions and the advocacy components of photovoice. In 2016, advocacy efforts included a public exhibit during World Refugee Day including media coverage.^37^ In 2019, a presentation of data occurred for a public audience in a university setting.

Temporal changes were captured in several ways. The interviews that occurred before the first photovoice meeting in 2019 were designed to capture individual demographic changes and perspectives on changes since 2016. The first portion of the first photovoice meeting in 2019 was dedicated to open-ended questions designed explicitly to facilitate discussion about changes since we last met with the women in 2016. Finally, the photovoice groups in 2019 explicitly asked participants to share and discuss photos of their lives to allow the opportunity for participants and researchers to reflect, compare, and contrast changes over time together.

### Data analysis

Analysis procedures aligned with the study aim of conducting a qualitative participatory examination of experiences of health and integration for this specific population of women in their context over time. They commenced with a participatory approach in that participants, as opposed to the researchers, chose which photos they wished to share and discuss with the group. Data were transcribed verbatim and first analyzed independently by the authors, limiting the focus to the photos and accompanying texts chosen by participants. Data were organized with stories from transcripts matched with the photo that sparked the discussion, so both visual and textual data were included in the analysis.

Textual data from 2016 were analyzed using inductive qualitative analysis with an ethnographic approach, including line by line open coding followed by an iterative process of coding to align with the research goals, narrowing it down, and grouping into overarching themes and subthemes.^38^ Textual data from 2019 were analyzed using a combination of inductive and deductive coding. The deductive portion used codes developed from the 2016 results that we wanted to revisit, but were used as a guide allowing evolution for change.^30^ The inductive portion used line by line open coding to capture brand new emergent data in 2019 followed by axial coding, a qualitative research technique to link data together to develop overarching themes and subthemes.^39^ Finally, the photos, accompanying texts, themes, and subthemes from 2016 and 2019 were arranged for comparing, contrasting, and final examination of the full data set. We worked as a team throughout the analysis process, benefiting from transdisciplinary perspectives from public health and international relations and discussing differences until there was consensus.

### Rigor and limitations

Several actions were taken to establish trustworthiness and data quality.^39,40^ Member checking was built into the photovoice sessions in an iterative manner to continually confirm accuracy and understanding of what the participants were saying in conjunction with their photos. This was critical when conducting photovoice through an interpreter and because we, as the researchers, have different cultural backgrounds and orientation from the participants. An audit trail of study design, data collection, analysis, and follow-up was compiled, so that the majority of processes from 2016 were replicated in 2019. Having two investigators from different disciplines allowed a high level of reflexivity, which challenged us to examine and address assumptions and biases brought to the research from our respective disciplines and previous experiences.^40^

There were also limitations to this study. As a qualitative study, findings cannot be generalized and instead are meant to offer deep contextual knowledge. A research team member was affiliated with the refugee resettlement agency, presenting increased risk of bias. This team member offered critical linguistic and cultural insight, but it was necessary to continually implement ongoing informed consent and education to ensure participants could distinguish between her role in the research versus the agency. Another limitation was lack of back translation, which can decrease trustworthiness.^41^

## Results

Using photovoice and semistructured interviews, this study illuminated experiences with nutrition and food security for Congolese women refugees over time. We captured data from participants when they were newly resettled refugees who had been in the United States for <2 years and followed up 3 years later. Two major themes were (1) access to food in contrast with availability of abundance of food in the United States and (2) concern about what constitutes healthy food in the United States contrasted with accessing healthy and culturally appropriate food in the United States. Results spanned both points in time and were not mutually exclusive, with more than one theme evident across years, photos, and accompanying stories.

### Access to food in contrast with availability of abundance of food in the United States

Women expressed concern about access to food at both points of data collection. Most participants were eligible for the U.S. Department of Agriculture (USDA) Supplemental Nutrition Assistance Program. In 2016, several expressed fear that they would not be able to provide food for their family without food stamps. DU summarized the overall fear of hunger faced by the women:

about welfare, I am going to talk about my own suffering, ….., before the welfare stop your Medicaid or your food stamp, they need to make sure you have been at that job at least five months so you have saved before they stop it. Now you get a job today and tomorrow your Medicaid and your food stamp is stop, you do not even have any food in your house, then you go back to that hunger that I came with (Postinterview 2016).

Concern about access to food remained in 2019. A change we saw in 2019 was the role of social connections for enhancing food security as evidenced by two participants below.

Me with this mama beside me here, we are in the same apartment. Like now we don't all have a job. We are jobless, okay? We can meet every day, …..the closer we are, we meet…… Sometimes she cook and I don't have food stamp, I go eat at her house. We keep the culture going. We go just eat to the neighbor. (BM, FG2, 2019)

You come to my house ……But if I tell you I didn't eat you will give me……. You know when we are together like this, it gives us morale because here everybody has problem, you don't feel like you're alone. We guide together. We help each other and we see what to do, how to help, that's all that keeps us going. Uniting. (EM, FG2 2019).

This contrasted with several women taking pictures at both points in time to express their surprise and pleasure at the abundance of food in the United States. In 2016, several women took pictures of canned food to show their surprise, and in 2019 a few participants took pictures of the food aisle in grocery stores.


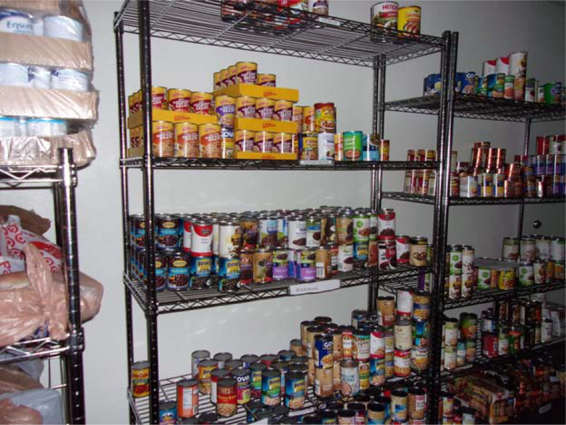

“Food cans—there is so much food in America—in cans, boxes, fresh—but not in Africa”.

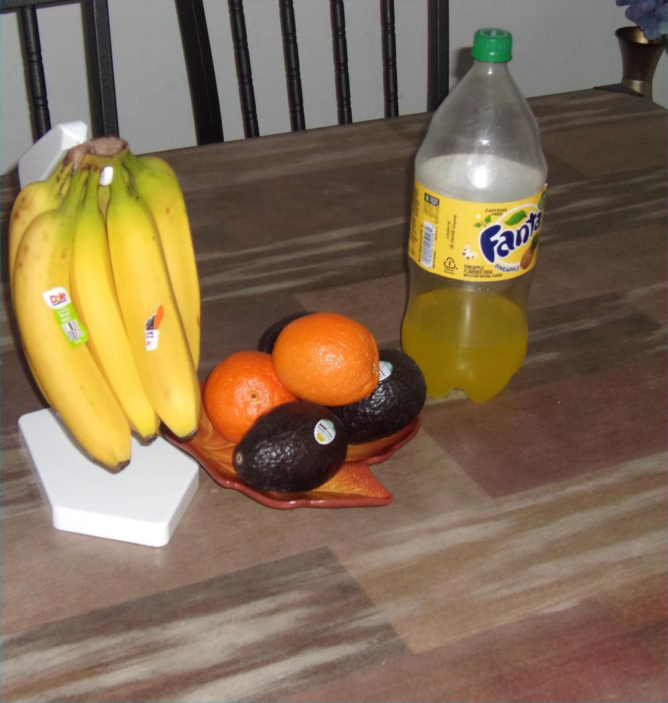

“I was one of the urban refugee,…living in a city back home, like an orange or an apple, it's very very expensive… but over here, oh my god, we buy all kind of fruit and you can afford it. It's like one dollar, a bunch of food. That makes me very happy as a Mama that I can provide healthy food to my kids.”

A finding that emerged in 2019 was an explicit reflection of previous food shortages and insecurity the participants faced in their home country and the refugee camps before they arrived in the United States. One participant in 2019 took a photo of beans cooking on the stove. She shared the first story to accompany the photo and CS chimed in.


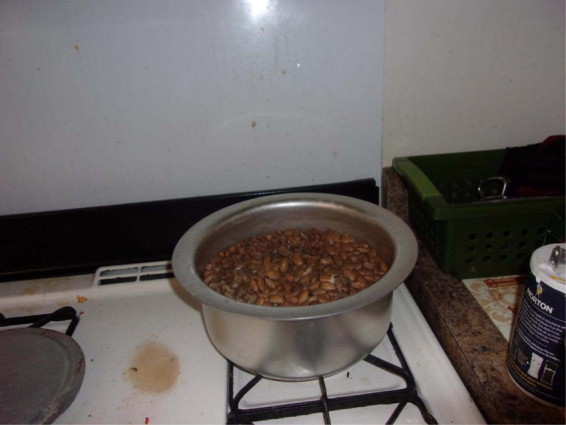

…so I took this picture … it just remind me of the poverty where we come from and how abundance we have (In Africa).You would cook a big pot like that and your neighbor, and your family members from the other…neighbors, like 20 people would come and ask a little bit, a little bit. You eat it with a lot of people, little with a lot of people. But right now I'm eating it alone.

EM explained her observations on how she felt life had changed since they came to America,

Our life has changed since we came here because honestly…. And if the visitor come, you just squeeze him there….. Especially food, ….. And we're like, oh, get away, get away. If there is a little food that you get on the table, we'll fight over it (Focus group 2, 2019).

Food security in connection with the support of a case manager and the refugee resettlement agency was mentioned as one participant shared her perspective on how their life was better in many ways in the United States:

And here you have a lot of hospital, a lot of case worker left and right. You can go to hospitals and ask for case worker. They will give you like a food pantry type of thing… Will give you a place to go pick up food. Or (refugee resettlement agency) will give you a address, you go get food. (DU, FG2 2019)

### Concern about what constitutes healthy food in the United States contrasted with accessing healthy food and culturally appropriate food in the United States

Over both points in time, several women took pictures of the Congolese food served during the meeting and chose those photos to express their joy at being able to find their cultural food.


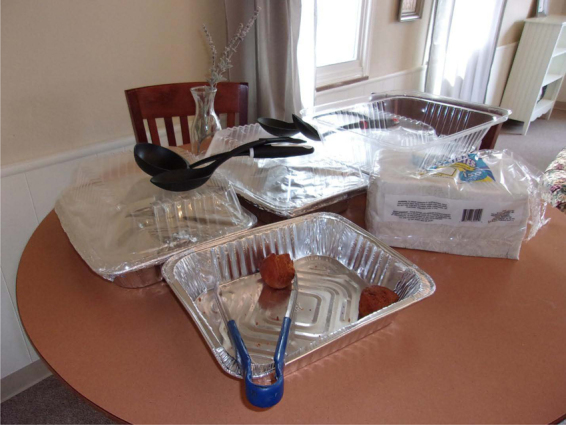

This food makes me happy because when we were in Africa we never know we are going to find our own food, like, our cultural appropriate food here in America. But you see we find it. We thought we were going to be eating from the cans… We go buy it and cook it.

What stood out in 2016 was the clear knowledge and intention to eat healthy food, particularly on the advice of their doctors. However, this was accompanied by uncertainty about what constituted healthy food in America and about how to access Congolese food that they knew to be healthy in terms of both availability and affordability. NN took a picture of a green vegetable and dried fish to make her point.


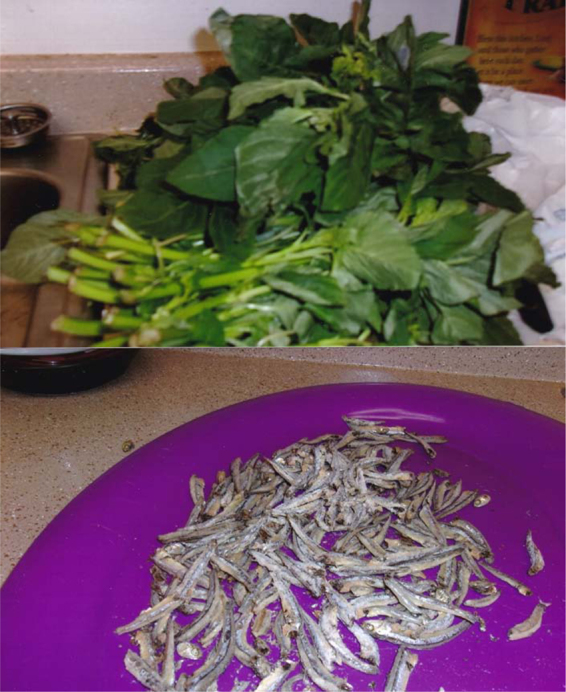

That this vegetable, one of the vegetables from Africa it is called Dodo. They have obligated me at the doctor to eat a lot of vegetables from home that I know, but it is very, very hard to find in this country. He … told me vegetables, and this is what I know vegetable is, …, but it is so hard to find, … I have to take them because I am pregnant. Some days we find them and some days we do not…. It's a dry fish, it is a very good nutrition that I know, but the purpose of taking the picture, it is so rare we cannot find it here. It is a dry fish but it is very nutritious and very good for you, it always expensive or hard to find it.)

By 2019, participants demonstrated noticeable improvements in being able to access healthy food in the United States. Participants chose to share photos showing preferences for fresh food versus packaged food that led to discussions about the health effect of such food. MS took a picture of a grocery aisle with fresh food,

This was just a relief when I saw them supermarkets when we get here, because we were told a lot. We were told a lot that we gonna be eating can food. Can food was what we thought we gonna be eating when we're here. But now we're so relieved. When I was relieved when I get to new supermarket and see that there's also fresh food, fresh vegetable.

Data also noted knowledge and concerns about the quality and nutritional value of food in the United States, including fear of chemical or hormonal additives to food and the culture of fast food. FN took a picture of a packaged chicken,

So this one make me sad because back home we take the real chicken and kill it and cook it. But this one because we heard a lot and I think they say they put medication in it to make it big…. And maybe those medication are not good for us. That make me sad, ‘cause I like chicken…. In Africa, it's natural. There's medication. Like milk. They add water. It's not natural. Nothing is natural here. That make me sad. I just put milk. I just had to add sugar in order to drink it.

MS took a picture of her kids to highlight her concern about the culture of fast food influencing her children.


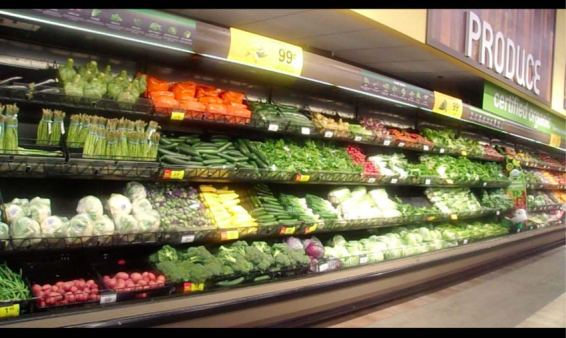



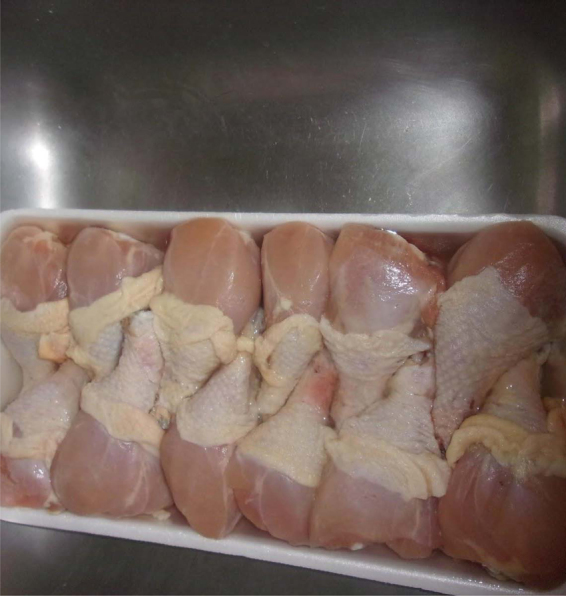

…those are my kids, right? But because they been here for couple years, even if I cook fufu and rice and bean like our traditional food, these two kid here won't eat…. They only wants food like McDonald or burger. I don't know. They are kid. They have catch up on American kids. But I cannot afford to buy them McDonald every day or every other day….

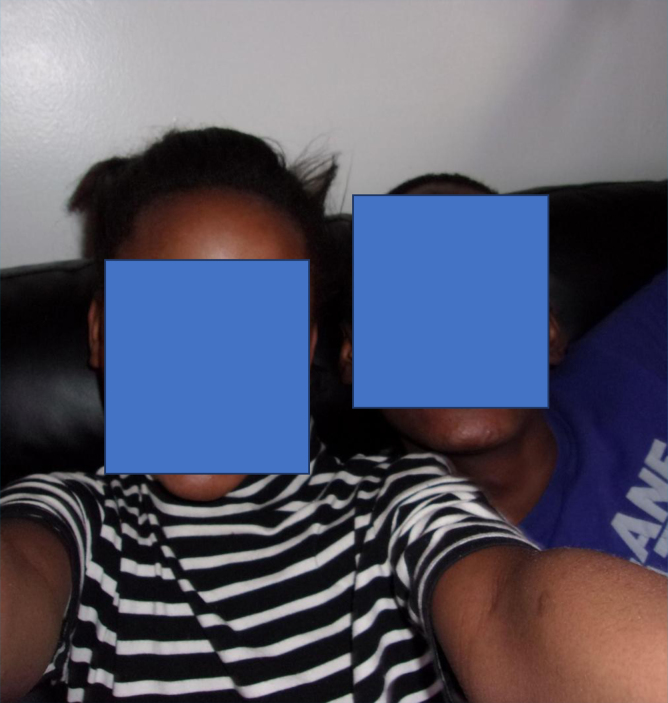


## Discussion

Congolese refugees have been resettling in the United States for decades with a sharp increase over the past 5 years. Yet, there is limited evidence to guide health promotion and care tailored for this population and less focused specifically on women. To our knowledge, this is the first longitudinal study to systematically examine health experiences of Congolese refugee women in a midwestern, U.S. city. Furthermore, there are gaps in the literature for both examination of the unique requirements of women and the distinctive needs related to nutritional health promotion and food security support for this population. Congolese women enter the country facing health inequities related to language barriers, trauma history, access to employment and health insurance, and racism in the United States,^4,5^ so such evidence is an imperative tool for public health and health care providers, as well as social service and refugee resettlement agencies to help build health equity and reduce health disparities.

Findings revealed assets of participant knowledge and attitudes recognizing and valuing healthy culturally familiar food supporting previous findings of critical health literacy assets that can be validated to enhance care and support for this population.^13^ This included noting preferences for fresh food over processed food, recognition of the health implications of mass food production, adding chemicals to food, and awareness and concern about the negative impacts of negative dietary acculturation such as their children preferring McDonald's to traditional foods. Participants also shared their perception that grocery stores in the United States lack culturally familiar choices. Our study showed a preference for traditional foods and understanding of them as fresher and healthier than the “American” diet, which aligns with the limited similar work conducted with this population in the United States so far.^29^

Our findings aligned with previous research with Congolese refugee women in a U.S. city,^19,27,28^ to reveal that access to food, both in general and to culturally familiar food, was a concern for the participants and confirmed that this concern remained several years past the initial resettlement period. Providing a longitudinal perspective, our research suggested that overtime, participants advanced their knowledge and skills in finding culturally familiar ingredients for traditional foods they felt were healthy. This represented an improvement over 2016 when some women lamented, they were advised by their doctor to “eat healthy,” but were struggling to find affordable culturally suitable food. This supports the finding of overall assets in critical health literacy for Congolese refugees,^12^ and on assets of existing knowledge of risks from “Westernized” diets,^14^ and offers the additional evidence for a woman-specific study. Health care providers and public health educators can improve approaches to care that strive for health equity by building on this existing knowledge and desire for healthy food including tailored education on accessing culturally familiar foods in the U.S. context at an affordable rate. Although it is important not to “romanticize or overemphasize resilience (Wachter, 2016. p. 885),” our research showed examples of assets and growth that are critical to explore with participants, honor, and recognize to advocate for health equity for this population since a deficit-based approach is often the default in serving Congolese refugee women.^6,9,13^

An additional asset of relying on each other for food and money when they fell short emerged by 2019, indicating a network of social support within the community of other Congolese refugee women. Such social networks can be a focal point for health care advocates and public health professionals to build upon when conducting education and support for this population. This has long been confirmed in the literature on the general population of refugees elsewhere in the world^42–44^ as well as more recently with studies including Congolese refugees and Congolese refugee women in the United States.^6,11,20^ Our findings add to the limited body of literature specifically focusing on Congolese women refugees in the U.S. context and also add the specific example of social support as a critical component for a financial safety net linking to food security.

Our findings also demonstrated a priority risk for food security, which is inextricably linked to the data showing that nearly all participants (90%) were not employed in 2019. This was a notably negative change over the course of the 3-year study period. Participants continued to face structural barriers to English language acquisition and the resulting negative ripple effect that has on jobs, economic stability, and ultimately food security. This was not surprising considering the disparity of higher food insecurity for African refugees in the United States estimated between 53% and 85%.^27,28^ However, it is a red flag for resettlement agencies and other providers to note the opportunity for intervention early in the resettlement process to impact the multiple structural determinants of English language acquisition, access to safe consistent employment, and economic access needed to maintain food security. In addition, the data showing reflection of prior food shortages such as the photo and story of the beans alongside data must be factored into to care and support for this population. Relying solely on the epidemiological data showing high rates of overweight, obesity, and hypertension for this population without a more comprehensive sociohistorical picture of prior and current food security can be detrimental.^22^ Other researchers have shown links between persistent food insecurity and higher risk for chronic diseases such as obesity and hypertension for refugees resettled in developed countries from other countries and regions such as Cambodia, the Middle East, and North Africa,^45,46^ and our results suggest this should also be explored more deeply for Congolese refugee women in the U.S. context.

## Conclusion

This qualitative inductive study aimed to broadly explore health and integration experiences of Congolese refugee women through photovoice and interviews over the time period from 2016 to 2019, revealing contextual data that can be considered by health care providers and public health professionals to improve nutritional health promotion and food security support for this population. Health care, social service, and resettlement service providers can contribute to building health equity for Congolese refugees in the United States when they shift to an assets and strength-based approach. This study highlights strengths bases of nutritional knowledge, attitudes, and skills as well as a strong social network aiding food security demonstrated by the Congolese refugee women in the study. Simultaneously, a clear and rather urgent risk of food insecurity linked to barriers to employment was revealed as was sociohistorical reflection on living with food shortages before migration to the United States. Overlooking these nuanced structural barriers may lead to providers offering subpar care that perpetuates health inequities for this population. We conclude by echoing Adekeye et al.'s (2014) call for advocacy for more research on culturally relevant health supports for African immigrants and expand the recommendation for more studies like ours to investigate health needs of refugees by country of origin to seize opportunities to promote health equity.

## Abbreviations Used

CBPR: community-based participatory research project
DRC: Democratic Republic of Congo
